# Di-μ-azido-κ^4^ *N*:*N*-bis­{aqua­[bis­(1*H*-benzimidazol-2-ylmeth­yl)amine]­copper(II)} dinitrate

**DOI:** 10.1107/S1600536811045909

**Published:** 2011-11-09

**Authors:** Yuan-yuan Luo

**Affiliations:** a Key Laboratory of Pesticide & Chemical Biology, Ministry of Education, College of Chemistry, Central China Normal University, Wuhan 430079, People’s Republic of China

## Abstract

In the centrosymmetric dinuclear title complex, [Cu_2_(N_3_)_2_(C_16_H_15_N_5_)_2_(H_2_O)_2_](NO_3_)_2_, the Cu^II^ ion is in a distorted octa­hedral coordination environment with long axial Cu—N_azide_ [2.821 (6) Å] and Cu—O_water_ [2.747 (5) Å] bonds as a result of the Jahn–Teller effect. Two symmetry-related azide ligands bridge in μ_2_-modes giving a Cu⋯Cu distance of 3.533 (2) Å. In the crystal, N—H⋯O and O—H⋯O hydrogen bonds link the components into a three-dimensional network. In addition, there are weak inter­molecular C—H⋯N hydrogen bonds and π–π stacking inter­actions with centroid–centroid distances ranging from 3.562 (2) to 3.974 (2) Å.

## Related literature

For the biological applications of polybenzimidazole metal coordination compounds, see: Liao *et al.* (2001 ▶); Girasolo *et al.* (2000 ▶); Young *et al.* (1995 ▶). For details of the Jahn–Teller effect, see: Brown *et al.* (1967 ▶). For the preparation of bis­[*N*-(benzimidazole-2-ylmeth­yl)] amine, see: Adams *et al.* (1990 ▶).
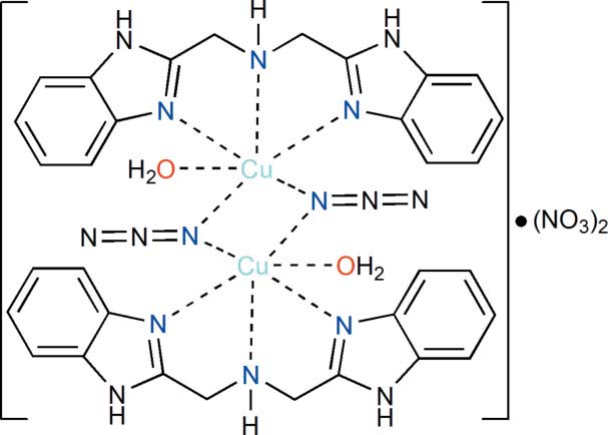


## Experimental

### 

#### Crystal data

[Cu_2_(N_3_)_2_(C_16_H_15_N_5_)_2_(H_2_O)_2_](NO_3_)_2_
*M* *_r_* = 925.85
Monoclinic, 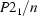

*a* = 11.1435 (10) Å
*b* = 14.1942 (12) Å
*c* = 12.6567 (11) Å
β = 112.046 (1)°
*V* = 1855.6 (3) Å^3^
*Z* = 2
Mo *K*α radiation
μ = 1.23 mm^−1^
*T* = 289 K
0.20 × 0.20 × 0.10 mm

#### Data collection

Bruker SMART APEX CCD diffractometer
Absorption correction: multi-scan (*SADABS*; Bruker, 2001 ▶) *T*_min_ = 0.792, *T*_max_ = 0.887
13705 measured reflections
3269 independent reflections
2307 reflections with *I* > 2σ(*I*)
*R*_int_ = 0.071

#### Refinement

*R*[*F* ^2^ > 2σ(*F* ^2^)] = 0.071
*wR*(*F* ^2^) = 0.171
*S* = 1.09
3269 reflections
286 parameters
6 restraints
H atoms treated by a mixture of independent and constrained refinement
Δρ_max_ = 0.78 e Å^−3^
Δρ_min_ = −0.49 e Å^−3^

### 

Data collection: *SMART* (Bruker, 2001 ▶); cell refinement: *SAINT* (Bruker, 2001 ▶); data reduction: *SAINT*; program(s) used to solve structure: *SHELXS97* (Sheldrick, 2008 ▶); program(s) used to refine structure: *SHELXL97* (Sheldrick, 2008 ▶); molecular graphics: *PLATON* (Spek, 2009 ▶); software used to prepare material for publication: *PLATON*.

## Supplementary Material

Crystal structure: contains datablock(s) global, I. DOI: 10.1107/S1600536811045909/lh5362sup1.cif

Structure factors: contains datablock(s) I. DOI: 10.1107/S1600536811045909/lh5362Isup2.hkl

Additional supplementary materials:  crystallographic information; 3D view; checkCIF report

## Figures and Tables

**Table 1:** Hydrogen-bond geometry (Å, °)

*D*—H⋯*A*	*D*—H	H⋯*A*	*D*⋯*A*	*D*—H⋯*A*
N1—H1*C*⋯O1	0.86 (2)	2.45 (4)	3.173 (7)	142 (5)
N1—H1*C*⋯O2	0.86 (2)	2.57 (3)	3.388 (8)	159 (6)
N3—H3*A*⋯O4^i^	0.83 (2)	2.02 (3)	2.825 (7)	162 (6)
N5—H5*A*⋯O1^ii^	0.86 (2)	2.02 (2)	2.868 (6)	171 (5)
O4—H4*A*⋯O2	0.84 (2)	2.06 (5)	2.810 (7)	149 (8)
O4—H4*B*⋯O3^iii^	0.83 (2)	2.21 (3)	3.022 (7)	166 (8)
C1—H1*B*⋯N8^i^	0.97	2.45	3.404 (9)	169

Symmetry codes: (i) 
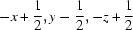
; (ii) 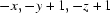; (iii) 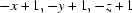.

**Fig. 1.**
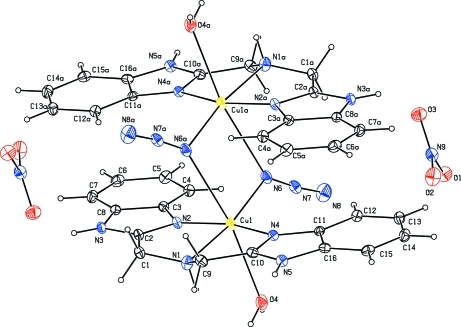
The molecular structure of (I) with displacement ellipsoids drawn at the 30% probability level (symmetry code (a): - x, 1 - y, - z).

**Fig. 2.**
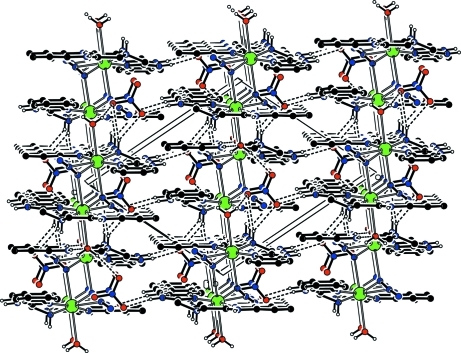
Part of the crystal structure of (I), showing the formation of the three-dimensional network by hydrogen bonds (dashed lines). Hydrogen atoms not involved in the motif have been omitted for clarity.

## supplementary crystallographic information

### Comment

Polybenzimidazole (Polybzim) metal coordination compounds have been often used to mimic some biological activities, such as superoxide dismutase (Liao *et al.*, 2001), DNA probe (Girasolo *et al.*, 2000), alkaline phosphatase (Young *et al.*, 1995). In this paper, we report the crystal structure of a polybzim copper complex (I), ([Cu(IDB)(µ-N_3_).H_2_O]_2_.(NO_3_)_2_), formed by bis[*N*-(benzimidazol-2-ylmethyl)]amine, sodium azide and copper nitrate in 95% methanol solution.

In (I), the long Cu—N6a (2.820 (5) Å, symmetry code (a): - *x*, 1 - *y*, - *z*) and Cu—O4 (2.747 (4) Å) bond distances are due to the Jahn-Teller effect (Brown *et al.*, 1967). In the centrosymmetric dinuclear complex, the unique Cu^II^ ion is in a distorted octahedral coordination environment. Two azide ligands bridgein µ_2_– modes giving a Cu···Cu distance of 3.533 (2)Å. In the crystal, cations and nitrate anions are linked into a three-dimensional network (Fig.2) by a combination of N—H···O, O—H···O and weak C—H···O hydrogen bonds (Table 2). In addtion, π–π stacking interactions with centroid to centroid distances ranging from 3.562 (2)Å to 3.974 (2) Å are also observed.

### Experimental

All the reagents and solvents were used as obtained without further purification. Bis(benzimidazol-2-yl-methyl) amine (IDB) was prepared according to the method described by Adams *et al.* (1990). IDB (0.277 g, 1.0 mmol), NaN_3_ (0.065 g, 1.0 mmol) and Cu(NO_3_)_2_.3H_2_O (0.242 g, 1.0 mmol) was dissolved in 15 ml me thanol and stirred for half an hour. The resulting solution was filtered and the resulting solution was stand at room temperature for two weeks. Then blue block-shaped crystals of (I) suitable for for X-ray diffraction  were obtained at the bottom of the vessel.

### Refinement

H atoms bonded to C atoms were placed in ideal positions refined in a riding-model appoximation with C–H distances of 0.93Å (aromatic), 0.97Å (methylene) and with *U*_iso_(H) = 1.2*U*_eq_(C). H atoms bonded to N and O atoms were found in difference maps and refined with constraints of N—H = 0.86 (2)Å and O—H = 0.82 (2) Å, and *U*_iso_(H) = 1.2U_eq_(N) or 1.5U_eq_(O).

### Crystal data

**Table:**

[Cu_2_(N_3_)_2_(C_16_H_15_N_5_)_2_(H_2_O)_2_](NO_3_)_2_	*F*(000) = 948
*M**_r_* = 925.85	*D*_x_ = 1.657 Mg m^−^^3^
Monoclinic, *P*2_1_/*n*	Mo *K*α radiation, λ = 0.71073 Å
Hall symbol: -P 2yn	Cell parameters from 1625 reflections
*a* = 11.1435 (10) Å	θ = 2.3–19.7°
*b* = 14.1942 (12) Å	µ = 1.23 mm^−^^1^
*c* = 12.6567 (11) Å	*T* = 289 K
β = 112.046 (1)°	Block, blue
*V* = 1855.6 (3) Å^3^	0.20 × 0.20 × 0.10 mm
*Z* = 2	

### Data collection

**Table:**

Bruker SMART APEX CCD diffractometer	3269 independent reflections
Radiation source: fine focus sealed Siemens Mo tube	2307 reflections with *I* > 2σ(*I*)
graphite	*R*_int_ = 0.071
0.3° wide ω exposures scans	θ_max_ = 25.0°, θ_min_ = 2.1°
Absorption correction: multi-scan (*SADABS*; Bruker, 2001)	*h* = −13→13
*T*_min_ = 0.792, *T*_max_ = 0.887	*k* = −16→14
13705 measured reflections	*l* = −15→15

### Refinement

**Table:**

Refinement on *F*^2^	Primary atom site location: structure-invariant direct methods
Least-squares matrix: full	Secondary atom site location: difference Fourier map
*R*[*F*^2^ > 2σ(*F*^2^)] = 0.071	Hydrogen site location: inferred from neighbouring sites
*wR*(*F*^2^) = 0.171	H atoms treated by a mixture of independent and constrained refinement
*S* = 1.09	*w* = 1/[σ^2^(*F*_o_^2^) + (0.0796*P*)^2^ + 1.2353*P*] where *P* = (*F*_o_^2^ + 2*F*_c_^2^)/3
3269 reflections	(Δ/σ)_max_ = 0.001
286 parameters	Δρ_max_ = 0.78 e Å^−^^3^
6 restraints	Δρ_min_ = −0.49 e Å^−^^3^

### Special details

**Table:**

Geometry. All e.s.d.'s (except the e.s.d. in the dihedral angle between two l.s. planes) are estimated using the full covariance matrix. The cell e.s.d.'s are taken into account individually in the estimation of e.s.d.'s in distances, angles and torsion angles; correlations between e.s.d.'s in cell parameters are only used when they are defined by crystal symmetry. An approximate (isotropic) treatment of cell e.s.d.'s is used for estimating e.s.d.'s involving l.s. planes.
Refinement. Refinement of *F*^2^ against ALL reflections. The weighted *R*-factor *wR* and goodness of fit *S* are based on *F*^2^, conventional *R*-factors *R* are based on *F*, with *F* set to zero for negative *F*^2^. The threshold expression of *F*^2^ > σ(*F*^2^) is used only for calculating *R*-factors(gt) *etc*. and is not relevant to the choice of reflections for refinement. *R*-factors based on *F*^2^ are statistically about twice as large as those based on *F*, and *R*- factors based on ALL data will be even larger.

### Fractional atomic coordinates and isotropic or equivalent isotropic displacement parameters (Å^2^)

**Table:**

	*x*	*y*	*z*	*U*_iso_*/*U*_eq_	
Cu1	0.11004 (7)	0.50588 (5)	0.14302 (5)	0.0427 (3)	
C1	0.0889 (6)	0.3158 (4)	0.2197 (5)	0.0482 (15)	
H1A	0.0034	0.2917	0.1748	0.058*	
H1B	0.1275	0.2757	0.2859	0.058*	
C2	0.1714 (5)	0.3173 (4)	0.1500 (4)	0.0393 (13)	
C3	0.2744 (5)	0.3736 (4)	0.0492 (4)	0.0376 (13)	
C4	0.3333 (5)	0.4286 (4)	−0.0078 (4)	0.0438 (14)	
H4	0.3273	0.4939	−0.0082	0.053*	
C5	0.4012 (6)	0.3829 (5)	−0.0638 (5)	0.0514 (16)	
H5	0.4397	0.4181	−0.1044	0.062*	
C6	0.4134 (6)	0.2848 (5)	−0.0609 (5)	0.0556 (17)	
H6	0.4618	0.2562	−0.0979	0.067*	
C7	0.3557 (6)	0.2304 (4)	−0.0050 (5)	0.0497 (15)	
H7	0.3623	0.1651	−0.0045	0.060*	
C8	0.2877 (5)	0.2751 (4)	0.0506 (4)	0.0396 (13)	
C9	−0.0342 (6)	0.4390 (4)	0.2785 (5)	0.0486 (15)	
H9A	−0.0233	0.4218	0.3557	0.058*	
H9B	−0.1107	0.4076	0.2260	0.058*	
C10	−0.0471 (5)	0.5443 (4)	0.2630 (4)	0.0422 (13)	
C11	−0.0245 (5)	0.6839 (4)	0.2032 (4)	0.0352 (12)	
C12	0.0091 (5)	0.7629 (4)	0.1561 (4)	0.0425 (14)	
H12	0.0600	0.7585	0.1125	0.051*	
C13	−0.0366 (5)	0.8486 (4)	0.1771 (5)	0.0462 (14)	
H13	−0.0172	0.9028	0.1455	0.055*	
C14	−0.1105 (6)	0.8561 (5)	0.2438 (5)	0.0525 (16)	
H14	−0.1379	0.9153	0.2568	0.063*	
C15	−0.1442 (5)	0.7788 (4)	0.2909 (5)	0.0479 (15)	
H15	−0.1941	0.7840	0.3353	0.058*	
C16	−0.0999 (5)	0.6915 (4)	0.2692 (4)	0.0415 (14)	
N1	0.0799 (5)	0.4121 (4)	0.2551 (4)	0.0534 (13)	
H1C	0.144 (4)	0.429 (5)	0.315 (3)	0.064*	
N2	0.2008 (4)	0.3982 (3)	0.1129 (4)	0.0413 (11)	
N3	0.2202 (5)	0.2429 (3)	0.1156 (4)	0.0429 (11)	
H3A	0.214 (6)	0.1862 (17)	0.129 (5)	0.051*	
N4	0.0095 (4)	0.5898 (3)	0.2030 (3)	0.0363 (10)	
N5	−0.1132 (4)	0.6021 (3)	0.3045 (4)	0.0425 (11)	
H5A	−0.150 (5)	0.586 (4)	0.350 (4)	0.051*	
N6	0.1104 (5)	0.5834 (4)	0.0151 (4)	0.0523 (13)	
N7	0.1896 (6)	0.6430 (4)	0.0265 (4)	0.0521 (13)	
N8	0.2602 (7)	0.7025 (5)	0.0279 (6)	0.091 (2)	
N9	0.3202 (6)	0.4259 (4)	0.5493 (5)	0.0575 (14)	
O1	0.2058 (5)	0.4516 (4)	0.5211 (4)	0.0719 (13)	
O2	0.3693 (5)	0.4251 (4)	0.4759 (4)	0.0842 (16)	
O3	0.3826 (5)	0.4022 (4)	0.6480 (4)	0.0759 (14)	
O4	0.3352 (5)	0.5660 (4)	0.3111 (4)	0.0681 (13)	
H4A	0.335 (6)	0.541 (6)	0.371 (4)	0.102*	
H4B	0.410 (3)	0.568 (6)	0.313 (6)	0.102*	

### Atomic displacement parameters (Å^2^)

**Table:**

	*U*^11^	*U*^22^	*U*^33^	*U*^12^	*U*^13^	*U*^23^
Cu1	0.0529 (5)	0.0355 (5)	0.0500 (4)	0.0068 (3)	0.0310 (4)	0.0091 (3)
C1	0.054 (4)	0.038 (4)	0.055 (4)	0.001 (3)	0.023 (3)	0.011 (3)
C2	0.044 (3)	0.032 (3)	0.044 (3)	0.002 (3)	0.019 (3)	0.007 (2)
C3	0.037 (3)	0.038 (3)	0.038 (3)	0.001 (3)	0.015 (3)	0.000 (2)
C4	0.040 (3)	0.043 (4)	0.051 (3)	−0.003 (3)	0.019 (3)	−0.001 (3)
C5	0.050 (4)	0.056 (4)	0.055 (4)	−0.007 (3)	0.028 (3)	−0.007 (3)
C6	0.049 (4)	0.065 (5)	0.053 (4)	0.005 (3)	0.019 (3)	−0.012 (3)
C7	0.044 (4)	0.040 (4)	0.060 (4)	0.004 (3)	0.015 (3)	−0.008 (3)
C8	0.037 (3)	0.036 (3)	0.039 (3)	0.001 (3)	0.007 (3)	−0.001 (2)
C9	0.044 (4)	0.054 (4)	0.053 (3)	0.000 (3)	0.023 (3)	0.013 (3)
C10	0.041 (3)	0.048 (4)	0.039 (3)	0.001 (3)	0.016 (3)	0.005 (3)
C11	0.032 (3)	0.036 (3)	0.033 (3)	0.002 (2)	0.008 (2)	−0.004 (2)
C12	0.044 (3)	0.042 (4)	0.046 (3)	−0.003 (3)	0.022 (3)	−0.002 (3)
C13	0.040 (3)	0.041 (4)	0.058 (4)	−0.003 (3)	0.019 (3)	0.000 (3)
C14	0.052 (4)	0.043 (4)	0.062 (4)	0.003 (3)	0.021 (3)	−0.009 (3)
C15	0.041 (4)	0.062 (4)	0.042 (3)	0.006 (3)	0.016 (3)	−0.010 (3)
C16	0.034 (3)	0.050 (4)	0.037 (3)	0.001 (3)	0.009 (3)	0.001 (3)
N1	0.059 (4)	0.047 (3)	0.067 (3)	−0.008 (3)	0.039 (3)	−0.001 (3)
N2	0.044 (3)	0.033 (3)	0.049 (3)	0.005 (2)	0.020 (2)	0.008 (2)
N3	0.048 (3)	0.030 (3)	0.050 (3)	0.002 (2)	0.017 (2)	0.005 (2)
N4	0.036 (3)	0.038 (3)	0.038 (2)	0.001 (2)	0.018 (2)	0.0039 (19)
N5	0.043 (3)	0.048 (3)	0.046 (3)	0.003 (2)	0.027 (2)	0.003 (2)
N6	0.068 (4)	0.044 (3)	0.054 (3)	0.003 (3)	0.033 (3)	0.012 (2)
N7	0.064 (4)	0.053 (4)	0.053 (3)	0.016 (3)	0.037 (3)	0.013 (3)
N8	0.110 (6)	0.083 (5)	0.108 (5)	−0.030 (4)	0.071 (5)	−0.003 (4)
N9	0.072 (4)	0.054 (4)	0.060 (4)	−0.012 (3)	0.040 (3)	−0.005 (3)
O1	0.062 (3)	0.086 (4)	0.078 (3)	0.011 (3)	0.038 (3)	0.005 (3)
O2	0.107 (4)	0.084 (4)	0.100 (4)	0.007 (3)	0.083 (3)	0.009 (3)
O3	0.084 (4)	0.084 (4)	0.063 (3)	0.000 (3)	0.031 (3)	0.006 (3)
O4	0.072 (3)	0.048 (3)	0.084 (3)	0.014 (3)	0.029 (3)	−0.003 (2)

### Geometric parameters (Å, °)

**Table:**

Cu1—N2	1.947 (4)	C9—H9A	0.9700
Cu1—N6	1.959 (5)	C9—H9B	0.9700
Cu1—N4	1.972 (4)	C10—N4	1.323 (6)
Cu1—N1	2.062 (5)	C10—N5	1.336 (7)
Cu1—O4	2.747 (5)	C11—C12	1.386 (7)
Cu1—N6^i^	2.821 (6)	C11—N4	1.388 (6)
C1—N1	1.454 (8)	C11—C16	1.394 (7)
C1—C2	1.494 (7)	C12—C13	1.381 (8)
C1—H1A	0.9700	C12—H12	0.9300
C1—H1B	0.9700	C13—C14	1.388 (8)
C2—N2	1.327 (7)	C13—H13	0.9300
C2—N3	1.333 (7)	C14—C15	1.367 (8)
C3—C4	1.384 (7)	C14—H14	0.9300
C3—N2	1.394 (6)	C15—C16	1.398 (8)
C3—C8	1.405 (8)	C15—H15	0.9300
C4—C5	1.377 (7)	C16—N5	1.372 (7)
C4—H4	0.9300	N1—H1C	0.86 (2)
C5—C6	1.399 (9)	N3—H3A	0.83 (2)
C5—H5	0.9300	N5—H5A	0.86 (2)
C6—C7	1.360 (8)	N6—N7	1.191 (7)
C6—H6	0.9300	N7—N8	1.150 (8)
C7—C8	1.367 (8)	N9—O3	1.228 (6)
C7—H7	0.9300	N9—O2	1.243 (6)
C8—N3	1.385 (7)	N9—O1	1.243 (7)
C9—N1	1.460 (7)	O4—H4A	0.84 (2)
C9—C10	1.507 (8)	O4—H4B	0.83 (2)
			
N2—Cu1—N6	96.73 (19)	N4—C10—N5	112.2 (5)
N2—Cu1—N4	163.95 (17)	N4—C10—C9	121.1 (5)
N6—Cu1—N4	99.01 (19)	N5—C10—C9	126.7 (5)
N2—Cu1—N1	81.88 (19)	C12—C11—N4	131.3 (5)
N6—Cu1—N1	169.2 (2)	C12—C11—C16	121.0 (5)
N4—Cu1—N1	82.07 (19)	N4—C11—C16	107.6 (5)
N2—Cu1—O4	90.27 (17)	C13—C12—C11	116.8 (5)
N6—Cu1—O4	100.4 (2)	C13—C12—H12	121.6
N4—Cu1—O4	90.01 (16)	C11—C12—H12	121.6
N1—Cu1—O4	90.29 (18)	C12—C13—C14	122.0 (6)
N2—Cu1—N6^i^	83.48 (17)	C12—C13—H13	119.0
N6—Cu1—N6^i^	86.4 (2)	C14—C13—H13	119.0
N4—Cu1—N6^i^	94.31 (16)	C15—C14—C13	121.8 (6)
N1—Cu1—N6^i^	82.82 (18)	C15—C14—H14	119.1
O4—Cu1—N6^i^	171.27 (14)	C13—C14—H14	119.1
N1—C1—C2	107.3 (5)	C14—C15—C16	116.8 (5)
N1—C1—H1A	110.3	C14—C15—H15	121.6
C2—C1—H1A	110.3	C16—C15—H15	121.6
N1—C1—H1B	110.3	N5—C16—C11	106.6 (5)
C2—C1—H1B	110.3	N5—C16—C15	131.8 (5)
H1A—C1—H1B	108.5	C11—C16—C15	121.6 (5)
N2—C2—N3	112.6 (5)	C1—N1—C9	118.3 (5)
N2—C2—C1	120.6 (5)	C1—N1—Cu1	110.3 (3)
N3—C2—C1	126.8 (5)	C9—N1—Cu1	110.5 (4)
C4—C3—N2	131.1 (5)	C1—N1—H1C	114 (5)
C4—C3—C8	119.9 (5)	C9—N1—H1C	104 (4)
N2—C3—C8	109.0 (5)	Cu1—N1—H1C	98 (4)
C5—C4—C3	117.5 (6)	C2—N2—C3	105.3 (4)
C5—C4—H4	121.2	C2—N2—Cu1	113.3 (3)
C3—C4—H4	121.2	C3—N2—Cu1	140.5 (4)
C4—C5—C6	121.5 (6)	C2—N3—C8	108.2 (4)
C4—C5—H5	119.3	C2—N3—H3A	128 (4)
C6—C5—H5	119.3	C8—N3—H3A	123 (4)
C7—C6—C5	121.2 (6)	C10—N4—C11	106.3 (4)
C7—C6—H6	119.4	C10—N4—Cu1	112.8 (4)
C5—C6—H6	119.4	C11—N4—Cu1	140.9 (3)
C6—C7—C8	117.7 (6)	C10—N5—C16	107.3 (4)
C6—C7—H7	121.1	C10—N5—H5A	125 (4)
C8—C7—H7	121.1	C16—N5—H5A	127 (4)
C7—C8—N3	133.0 (6)	N7—N6—Cu1	122.0 (4)
C7—C8—C3	122.1 (5)	N8—N7—N6	174.1 (7)
N3—C8—C3	104.9 (5)	O3—N9—O2	121.2 (6)
N1—C9—C10	106.3 (4)	O3—N9—O1	120.0 (5)
N1—C9—H9A	110.5	O2—N9—O1	118.7 (6)
C10—C9—H9A	110.5	Cu1—O4—H4A	105 (5)
N1—C9—H9B	110.5	Cu1—O4—H4B	131 (5)
C10—C9—H9B	110.5	H4A—O4—H4B	109 (3)
H9A—C9—H9B	108.7		
			
N1—C1—C2—N2	−12.8 (7)	C8—C3—N2—C2	−0.2 (6)
N1—C1—C2—N3	169.1 (5)	C4—C3—N2—Cu1	13.8 (10)
N2—C3—C4—C5	179.7 (5)	C8—C3—N2—Cu1	−167.8 (4)
C8—C3—C4—C5	1.4 (8)	N6—Cu1—N2—C2	−152.5 (4)
C3—C4—C5—C6	−1.7 (9)	N4—Cu1—N2—C2	16.0 (9)
C4—C5—C6—C7	1.7 (9)	N1—Cu1—N2—C2	16.7 (4)
C5—C6—C7—C8	−1.4 (9)	O4—Cu1—N2—C2	107.0 (4)
C6—C7—C8—N3	−179.0 (6)	N6^i^—Cu1—N2—C2	−66.9 (4)
C6—C7—C8—C3	1.2 (8)	N6—Cu1—N2—C3	14.4 (6)
C4—C3—C8—C7	−1.3 (8)	N4—Cu1—N2—C3	−177.1 (6)
N2—C3—C8—C7	−179.9 (5)	N1—Cu1—N2—C3	−176.4 (6)
C4—C3—C8—N3	178.9 (5)	O4—Cu1—N2—C3	−86.1 (6)
N2—C3—C8—N3	0.3 (6)	N6^i^—Cu1—N2—C3	100.0 (6)
N1—C9—C10—N4	20.0 (7)	N2—C2—N3—C8	0.1 (6)
N1—C9—C10—N5	−160.1 (5)	C1—C2—N3—C8	178.4 (5)
N4—C11—C12—C13	−176.7 (5)	C7—C8—N3—C2	180.0 (6)
C16—C11—C12—C13	−0.5 (8)	C3—C8—N3—C2	−0.2 (6)
C11—C12—C13—C14	1.2 (8)	N5—C10—N4—C11	−1.4 (6)
C12—C13—C14—C15	−1.2 (9)	C9—C10—N4—C11	178.4 (5)
C13—C14—C15—C16	0.3 (9)	N5—C10—N4—Cu1	179.0 (4)
C12—C11—C16—N5	−178.7 (5)	C9—C10—N4—Cu1	−1.1 (6)
N4—C11—C16—N5	−1.8 (6)	C12—C11—N4—C10	178.5 (6)
C12—C11—C16—C15	−0.2 (8)	C16—C11—N4—C10	2.0 (6)
N4—C11—C16—C15	176.7 (5)	C12—C11—N4—Cu1	−2.2 (10)
C14—C15—C16—N5	178.4 (6)	C16—C11—N4—Cu1	−178.7 (4)
C14—C15—C16—C11	0.3 (8)	N2—Cu1—N4—C10	−11.6 (9)
C2—C1—N1—C9	153.2 (5)	N6—Cu1—N4—C10	156.8 (4)
C2—C1—N1—Cu1	24.7 (6)	N1—Cu1—N4—C10	−12.3 (4)
C10—C9—N1—C1	−156.2 (5)	O4—Cu1—N4—C10	−102.6 (4)
C10—C9—N1—Cu1	−27.8 (5)	N6^i^—Cu1—N4—C10	69.8 (4)
N2—Cu1—N1—C1	−23.7 (4)	N2—Cu1—N4—C11	169.1 (6)
N6—Cu1—N1—C1	59.6 (12)	N6—Cu1—N4—C11	−22.5 (6)
N4—Cu1—N1—C1	156.1 (4)	N1—Cu1—N4—C11	168.4 (6)
O4—Cu1—N1—C1	−113.9 (4)	O4—Cu1—N4—C11	78.1 (6)
N6^i^—Cu1—N1—C1	60.7 (4)	N6^i^—Cu1—N4—C11	−109.5 (6)
N2—Cu1—N1—C9	−156.3 (4)	N4—C10—N5—C16	0.3 (6)
N6—Cu1—N1—C9	−73.0 (12)	C9—C10—N5—C16	−179.6 (5)
N4—Cu1—N1—C9	23.4 (4)	C11—C16—N5—C10	0.9 (6)
O4—Cu1—N1—C9	113.4 (4)	C15—C16—N5—C10	−177.4 (6)
N6^i^—Cu1—N1—C9	−71.9 (4)	N2—Cu1—N6—N7	−97.0 (5)
N3—C2—N2—C3	0.1 (6)	N4—Cu1—N6—N7	86.2 (5)
C1—C2—N2—C3	−178.3 (5)	N1—Cu1—N6—N7	−178.9 (9)
N3—C2—N2—Cu1	171.5 (4)	O4—Cu1—N6—N7	−5.5 (5)
C1—C2—N2—Cu1	−6.9 (7)	N6^i^—Cu1—N6—N7	−180.0 (6)
C4—C3—N2—C2	−178.6 (6)		

Symmetry codes: (i) −*x*, −*y*+1, −*z*.

### Hydrogen-bond geometry (Å, °)

**Table:**

*D*—H···*A*	*D*—H	H···*A*	*D*···*A*	*D*—H···*A*
N1—H1C···O1	0.86 (2)	2.45 (4)	3.173 (7)	142 (5)
N1—H1C···O2	0.86 (2)	2.57 (3)	3.388 (8)	159 (6)
N3—H3A···O4^ii^	0.83 (2)	2.02 (3)	2.825 (7)	162 (6)
N5—H5A···O1^iii^	0.86 (2)	2.02 (2)	2.868 (6)	171 (5)
O4—H4A···O2	0.84 (2)	2.06 (5)	2.810 (7)	149 (8)
O4—H4B···O3^iv^	0.83 (2)	2.21 (3)	3.022 (7)	166 (8)
C1—H1B···N8^ii^	0.97	2.45	3.404 (9)	169.

Symmetry codes: (ii) −*x*+1/2, *y*−1/2, −*z*+1/2; (iii) −*x*, −*y*+1, −*z*+1; (iv) −*x*+1, −*y*+1, −*z*+1.
